# Dermatophytosis in a Healthy Adolescent: A Report of Terbinafine-Resistant Trichophyton indotineae Infection in Kuwait

**DOI:** 10.7759/cureus.84108

**Published:** 2025-05-14

**Authors:** Mohammad F Al Otaibi, Fahad AlSharhan, Fawziah AlRujaib, Reem AlQusaimi, Noria AlFadhel, Hanan Abdulwahab, Hanan Alajmi, Maryam AlHashel, Ayaa Alkhaleefa

**Affiliations:** 1 Dermatology, Abdulkareem Al-Saeed Dermatology Center, Kuwait City, KWT; 2 Dermatology, Jaber Al-Ahmed Hospital, Kuwait City, KWT; 3 Dermatology, Al-Amiri Hospital, Kuwait City, KWT

**Keywords:** cutaneous fungal infection, dermatophytosis, drug resistance, itraconazole, terbinafine, tinea, trichophyton indotineae

## Abstract

Dermatophytosis caused by *Trichophyton* species is a common fungal infection of the skin, hair, and nails. However, the emergence of antifungal-resistant *Trichophyton* strains has become an increasing challenge, leading to persistent and recurrent infections that are recalcitrant to treatment.

We present the case of a 19-year-old Yemeni male patient with a dermatophyte infection caused by a terbinafine-resistant *Trichophyton* species. The patient experienced symptoms for two weeks despite standard antifungal treatment. Fungal culture results showed the growth of *Trichophyton indotineae*. The patient was subsequently treated with topical clotrimazole 1% twice daily and luliconazole 1% cream at night, which led to the significant clinical improvement and complete resolution of the rash within two weeks.

This case highlights the growing concern of antifungal resistance in dermatophyte infections, particularly against terbinafine, one of the most commonly prescribed treatments. The increasing prevalence of resistant *Trichophyton* strains underscores the need for antifungal susceptibility testing in refractory cases and the consideration of alternative treatment strategies.

## Introduction

Dermatophytosis, also known as tinea, is the most common fungal infection worldwide, affecting approximately 25% of the general population. These infections are caused by dermatophytes that invade keratinized tissues, such as the skin, hair, and nails [1]. The causative organisms of these infections can be classified based on their ecological niche: anthropophilic species, which primarily infect humans; zoophilic species, which originate from animals; and geophilic species, which are found in soil. Globally, *Trichophyton rubrum* is the most frequently identified cause of dermatophytosis. However, *Trichophyton indotineae*, a newly identified species within the *Trichophyton mentagrophytes* complex, has recently emerged as the predominant pathogen in India and other parts of Asia. *T. indotineae* exhibits an anthropophilic instead of a zoophilic transmission pattern and has developed a high level of terbinafine resistance. Due to globalization, this new emerging pathogen has been isolated in many countries outside Asia, causing an epidemic of severe, recalcitrant dermatophytosis [2].

Herein, we present the case of a 19-year-old Yemeni male patient who presented with a two-month history of scaly, annular rash of the left hand and right cheek. The diagnosis of tinea caused by *T. indotineae* has been established based on fungal culture. This case highlights the challenges faced in diagnosing and managing patients with refractory tinea.

## Case presentation

A 19-year-old, previously healthy, Yemeni male patient presented to the dermatology outpatient department complaining of a two-month history of scaly, annular patches of rash on the left hand and right cheek and sideburn. These lesions appeared gradually and were increasing in size over the last two months. There was no oozing, burning sensation, pain, nor other constitutional symptoms associated with the rash. The patient never complained of similar lesions previously. No history of contact with people who have similar lesions was denoted. He was not on any regular medications or supplements and does not have any known drug allergies. There was no family history of similar rash or any other skin conditions. No pets are living in the house. There was no recent travel history. Overall, the patient has a reasonable general health and good hygiene.

**Figure 1:**
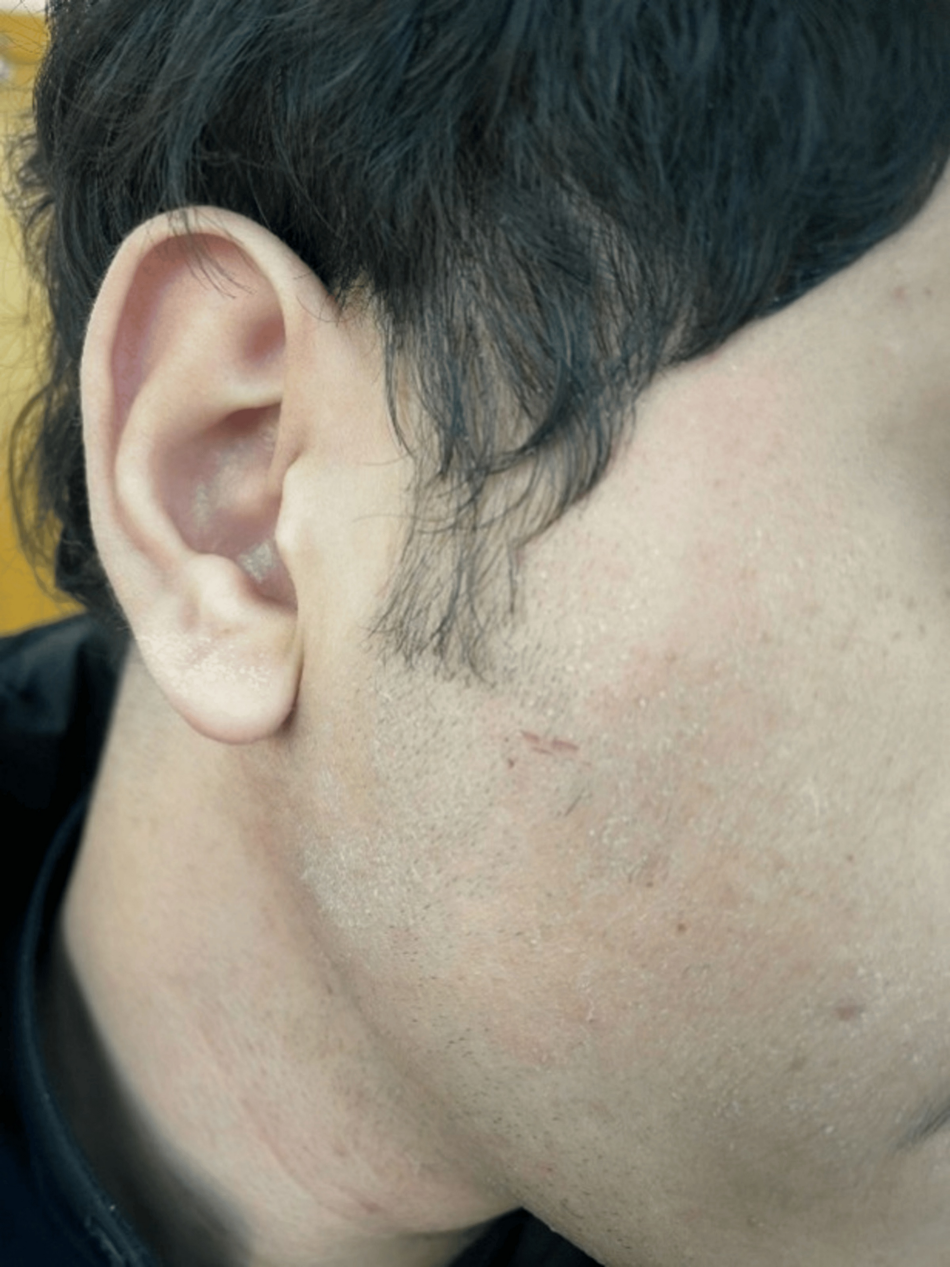
Clinical findings of dermatophytosis Physical examination showed annular, scaly, ill-defined patches involving the right cheek and sideburn.

**Figure 2:**
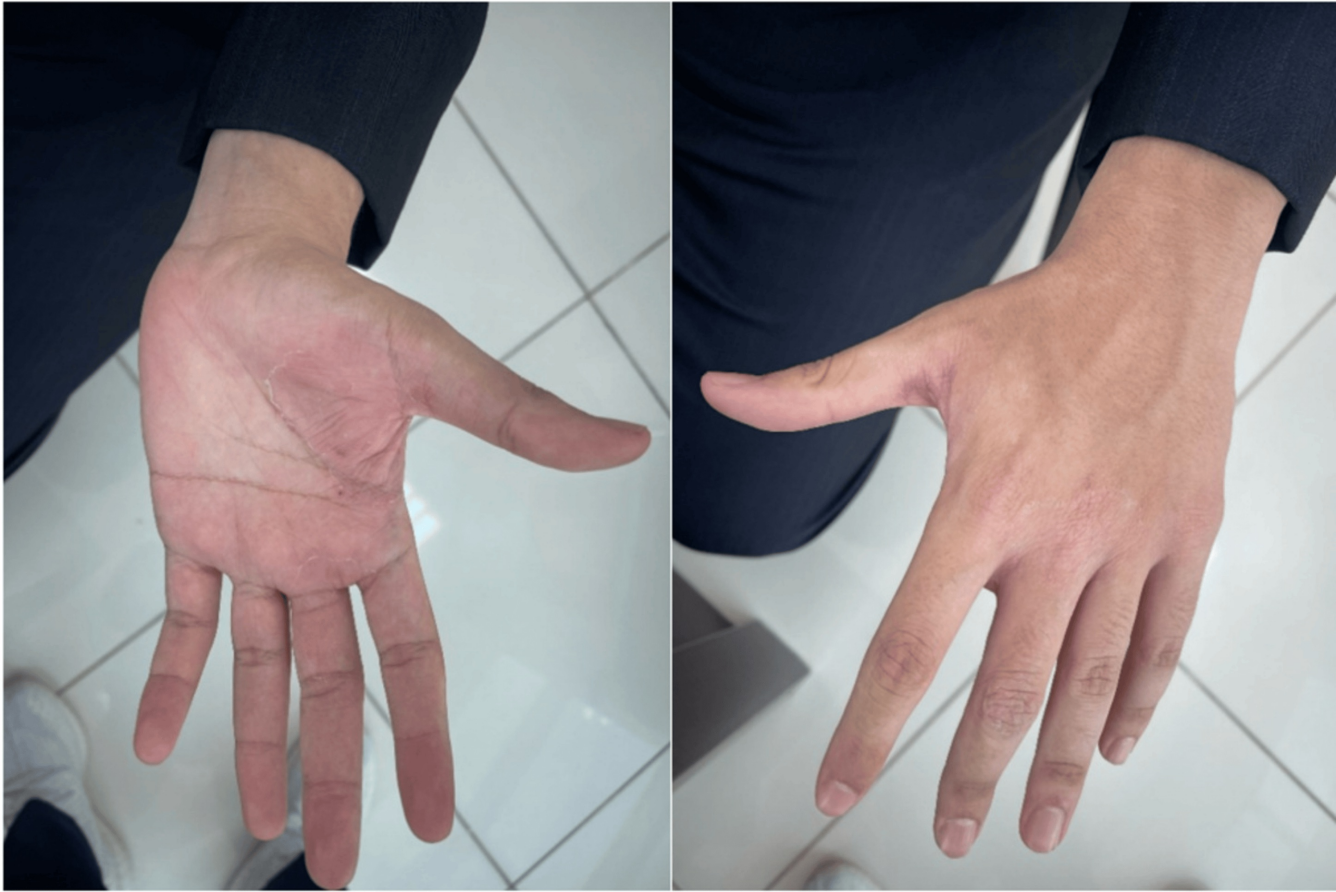
Clinical findings of dermatophytosis Physical examination showed similar well-demarcated and mildly erythematous lesions involving the left hand.

A keen examination of the face revealed annular patches covering the right cheek and sideburn, sparing the ear. These lesions were ill-defined, non-erythematous, and covered by fine scales (Figure 1). The left hand was also affected, as similar patches were observed on the area between the thumb and index finger on both the dorsum and palmar aspects, as well as the first two knuckles. These lesions however were well-demarcated and mildly erythematous (Figure 2). The patient is right-hand dominant and works a desk job, with no known occupational exposure to irritants or moisture. He was vitally stable and afebrile.

On the patient's first clinic visit, a fungal culture scraping was taken, and the patient was started on oral terbinafine at a dose of 250 mg once daily for two weeks, topical terbinafine 1% in the morning, topical miconazole at night, dexpanthenol cream twice daily, and desloratadine once daily at night to relieve the itchiness.

**Figure 3:**
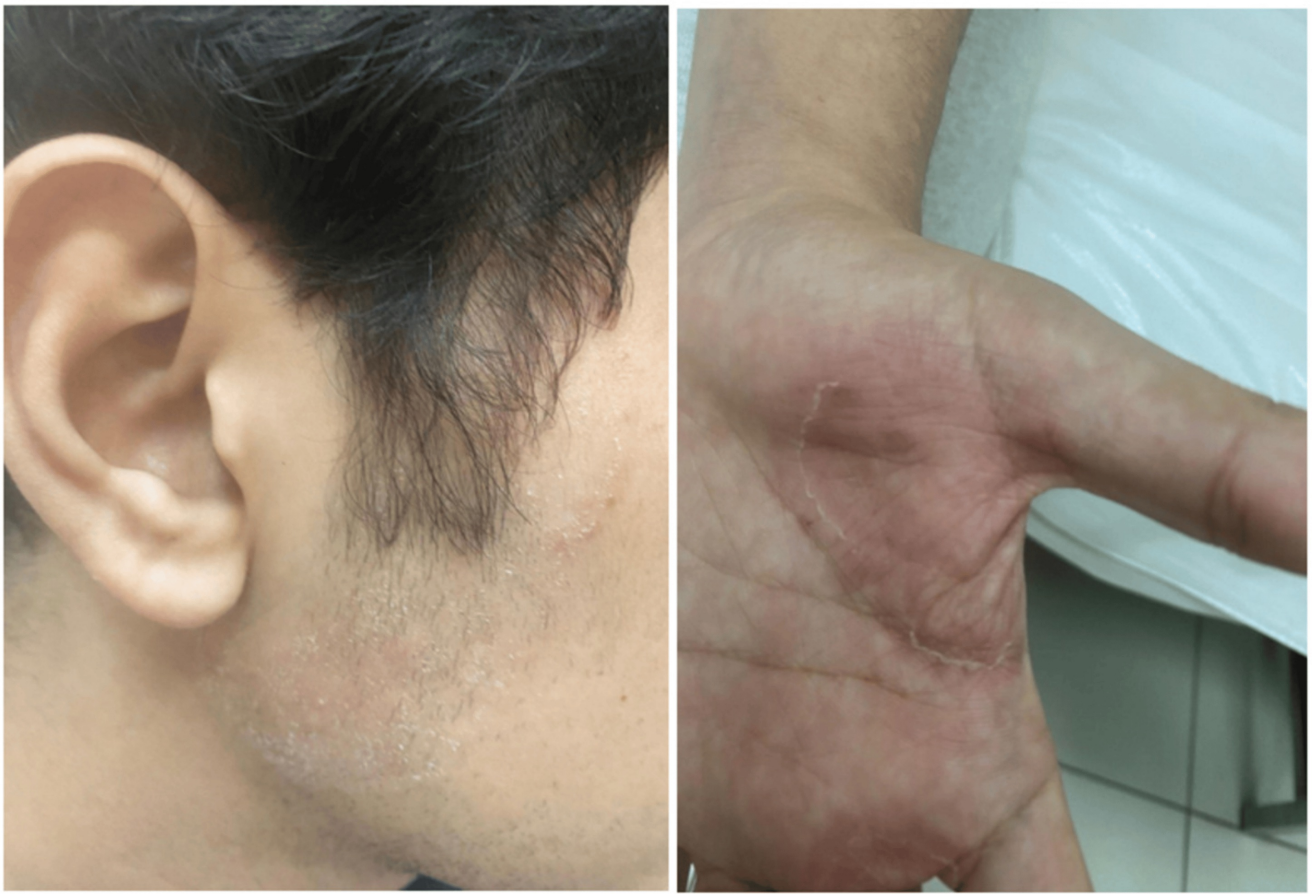
Follow-up after two weeks After two weeks of follow-up, the patient showed minimal improvement.

**Figure 4:**
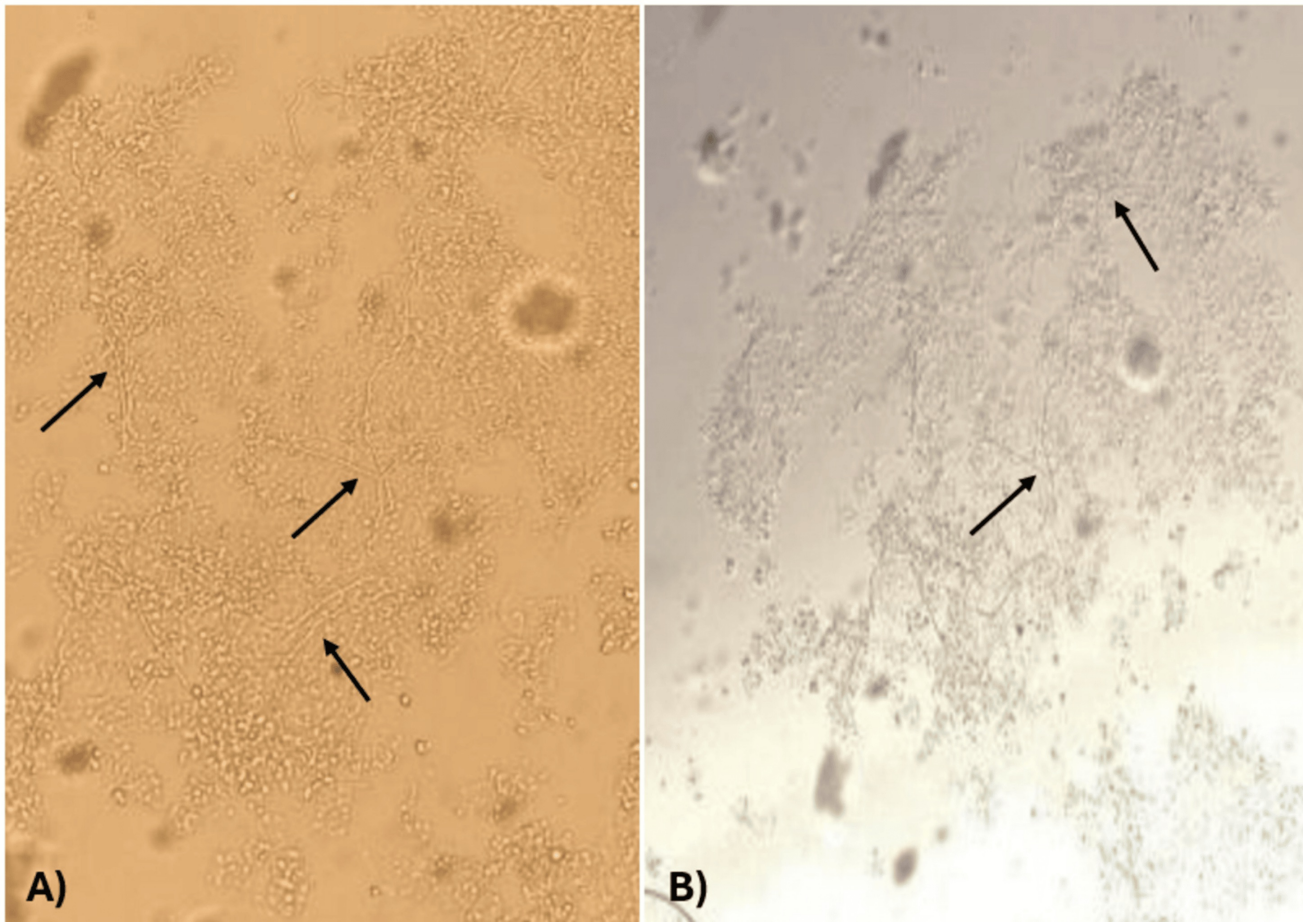
Microscopic findings from KOH preparation KOH mount (20%) of skin scrapings demonstrating septate, branching hyphae, and fungal spores (black arrows), indicative of a dermatophyte infection. (A) Fungal structures visualized on a dark background, enhancing contrast and highlighting delicate hyphae and spores. (B) Bright-field image showing the same fungal elements on a light background. KOH: potassium hydroxide

**Figure 5:**
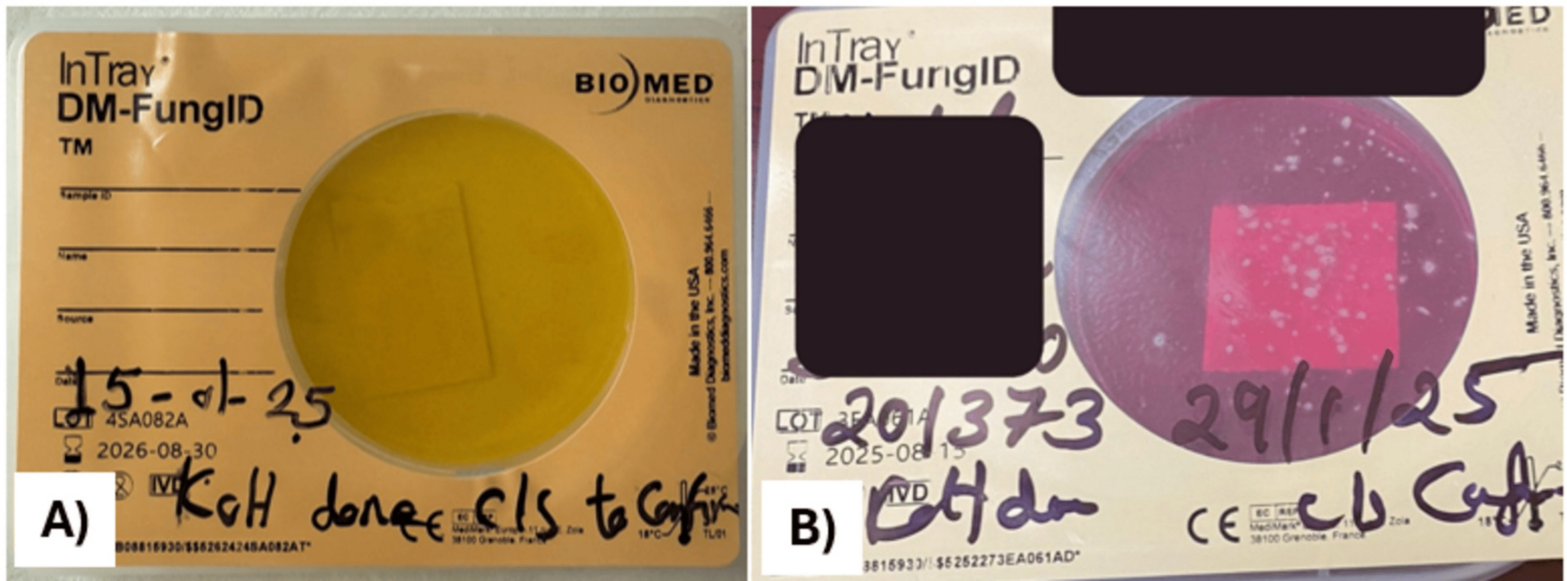
Dermatophyte test medium showing positive culture result Skin scrapings were inoculated onto dermatophyte test medium and incubated (A). After two weeks, the medium developed a red coloration, indicating dermatophyte growth (B).

**Figure 6:**
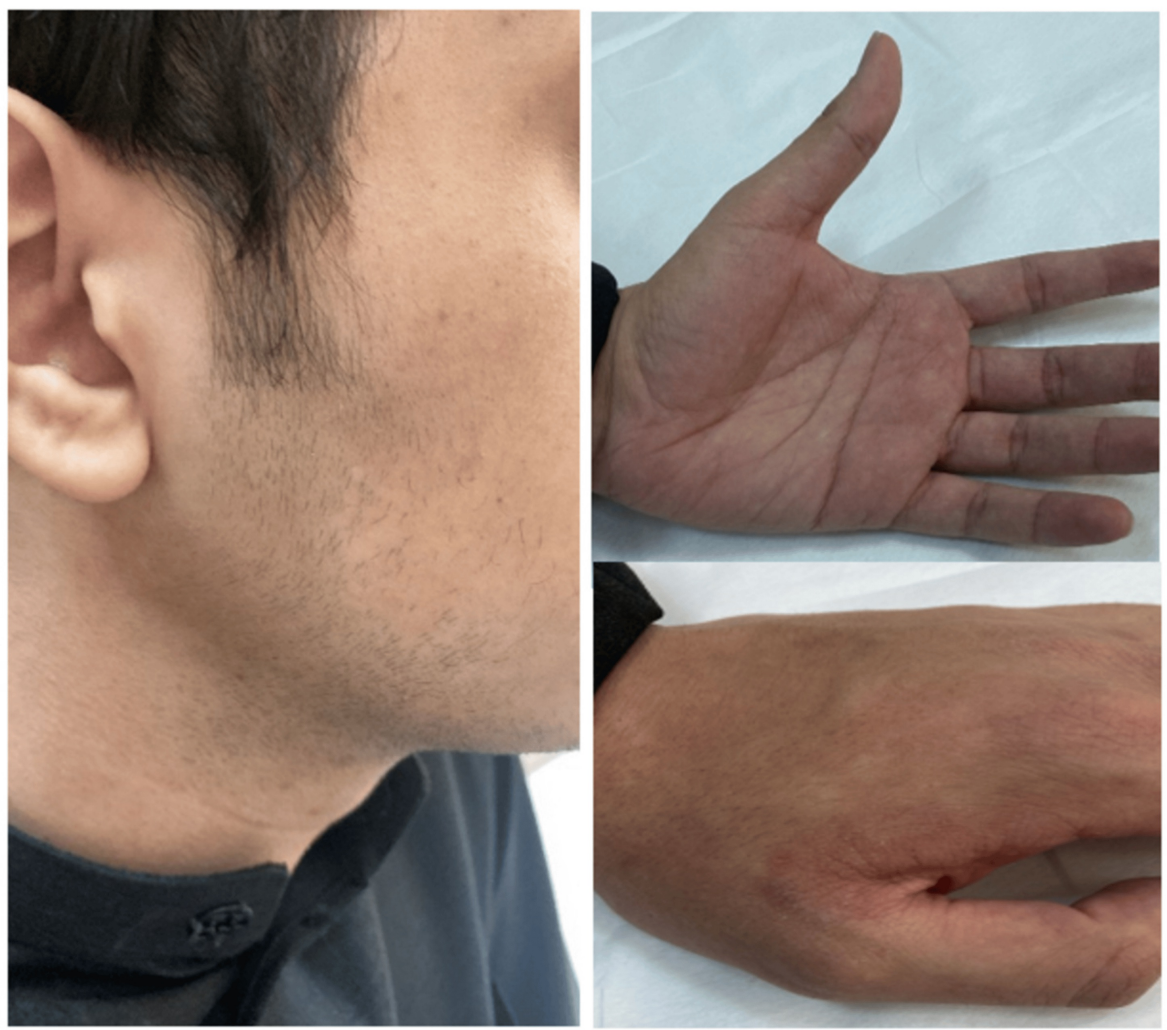
Follow-up after one month There was a marked improvement of the skin lesions, and the patient was satisfied with the results.

A follow-up after two weeks was scheduled to reassess the patient's condition and review culture results. The lesions showed minimal response to the initial treatment (Figure 3). Direct microscopic examination of skin scrapings was performed using a 20% potassium hydroxide (KOH) preparation, which revealed the presence of septate, branching hyphae along with spores, consistent with dermatophyte infection (Figure 4). Fungal culture was performed by inoculating skin scrapings onto a dermatophyte test medium. After two weeks of incubation at room temperature, the medium turned red, indicating alkaline metabolic byproducts, consistent with the presence of dermatophytes, supporting the diagnosis of *T. indotineae* (Figure 5). Susceptibility testing for terbinafine resistance was not performed due to resource limitations at our facility, but clinical resistance was suspected based on the poor initial response. Hence, medications were adjusted as follows: continue oral terbinafine for two more weeks, topical clotrimazole 1% twice daily, luliconazole 1% cream at night, and oral desloratadine. Follow-up after two weeks showed satisfactory results, as the rash had resolved completely and the patient was no longer complaining of itchiness (Figure 6).

## Discussion

Dermatophytes are the most common pathogenic filamentous fungi. These fungi typically infect the nails, skin, and hair, leading to various superficial dermatophytoses, including tinea capitis, onychomycosis, tinea corporis, and tinea pedis. The clinical presentation of superficial fungal skin infections is typically distinctive. Tinea corporis, tinea faciei, and tinea cruris often appear as solitary or multiple annular, scaly, erythematous macules and patches with central clearing that gradually expand. A characteristic feature of cutaneous dermatophytosis is the ring-within-a-ring pattern [3]. In rare cases, dermatophytes can invade deeper dermal tissues and even internal organs, particularly in immunocompromised individuals with congenital or acquired immunodeficiencies. If left untreated, these infections may progress to life-threatening conditions. Among dermatophytes, *T. rubrum* is the most frequently identified species worldwide, accounting for 50-90% of dermatophytosis. However, in recent years, India has seen a shift in the predominant pathogen from *T. rubrum* to the *T. mentagrophytes* complex, namely, *T. indotineae*. Additionally, the emergence of the drug-resistant dermatophyte, *T. indotineae*, has led to a significant rise in cases of recalcitrant and treatment-resistant infections in India [4]. Patients with these infections may present with double-edged or multi-edged concentric scaly rings. Large lesions featuring multiple closely grouped, centrifugally spreading patches with eczematous centers have also been reported [5]. These lesions are usually associated with severe pruritic and burning sensation [6].

Based on current knowledge, *T. indotineae* is primarily transmitted from person to person. Animal infections, or zoonotic sources, have been reported in only a few cases. It has been suggested that *T. indotineae* has undergone a process of obligate anthropization, meaning it has adapted to human hosts rather than animals. The species has shown a strong ability to thrive on the human epidermis and is easily spread through direct physical contact. However, indirect transmission can also occur through contaminated surfaces in shared environments, such as bathrooms, lavatories, and bed or body linens. Sexual transmission is also possible [7].

Terbinafine resistance in *T. indotineae* was first observed clinically when dermatophytosis failed to respond to treatment and worsened despite adequate oral antifungal therapy. Resistance mechanisms in dermatophytes arise from multiple factors, including the overexpression of drug efflux pumps and biofilm formation. However, one of the most frequently reported resistance mechanisms in *T. indotineae* involves point mutations in the squalene epoxidase (SQLE) gene, ERG1, which are linked to terbinafine resistance. These mutations modify the structure of the SQLE enzyme, reducing terbinafine's ability to bind effectively and thereby diminishing its antifungal activity. This resistance may be due to in vitro resistance or at least reduced in vitro sensitivity of the dermatophytes in question [8]. Additionally, azole resistance has been linked to the overexpression of the ATP-binding cassette (ABC) transporter gene multidrug resistance protein 3 (MDR3) and the amplification of the CYP51B gene, which encodes C14-α-demethylase [4]. There is a clear and frequently discussed temporal link between the widespread use of proprietary creams containing topical steroids and the recent surge in chronic, extensive, therapy-resistant dermatophytosis, an infection that was previously uncommon [1].

Key risk factors for recurrent dermatophytosis, aside from the use of topical steroids, include infrequent washing of clothes, lack of hygiene, overcrowding, work in a hot and humid environment, wearing occlusive or tight-fitting underwear, a family history of tinea, and sharing towels or bed linens. Moreover, factors that should not be overlooked include increased migration and the resurgence of tourism following the COVID-19 pandemic, which may have contributed to the spread of certain pathogens, including *T. indotineae*. From an immunological perspective, patients with chronic dermatophytosis often exhibit reduced interferon levels, decreased type 1 helper (Th1) and interleukin-17 (IL-17)-positive Th17 cells, and an impaired immune response, particularly in delayed-type hypersensitivity reactions observed in intradermal tests [9]. 

Although tinea is primarily diagnosed clinically, these cases underscore the importance of laboratory confirmation through culture, particularly in patients with treatment-resistant infections. Identifying the causative pathogen accurately is crucial for effective management. However, because *Trichophyton* species and other dermatophytes grow slowly, clinicians might be reluctant to request laboratory testing. Despite this, culture-based diagnosis remains the gold standard for confirming infection [10].

Mycological examination is crucial for a preliminary diagnosis before initiating treatment, as it helps minimize the risks associated with systemic antifungal therapy while remaining cost-effective. One commonly used method is the KOH examination, a simple bedside technique. This involves scraping keratin from the active border of a rash or an affected nail, preparing the sample with KOH, and examining it under a light microscope. The presence of branching septate hyphae confirms fungal infection. Although the accuracy of KOH examination depends on the experience of the technician, repeated testing can enhance diagnostic reliability [3].

Currently, antifungal susceptibility testing for dermatophytes is technically complex, which is why it is not routinely performed in clinical practice. However, accurately identifying dermatophyte strains at both the genus and species levels is crucial, not only to determine the probable source of infection but also to ensure targeted treatment. Phenotypic methods alone are insufficient for precise identification, making molecular techniques such as mass spectrometry and sequencing valuable additions [2].

The main challenge lies in the accurate identification of the species using molecular techniques or terbinafine sensitivity testing. This can be done through a simple breakpoint method or preferably a standardized microdilution, along with sequencing-based mutation analysis of the SQLE gene [11]. Therefore, sequencing of the internal transcribed spacer (ITS) region of ribosomal DNA is currently regarded as the gold standard for identifying *T. indotineae* [6].

Several conditions that present with annular lesions can resemble tinea corporis, making differential diagnosis essential. These conditions include pityriasis rosea, tinea versicolor, nummular eczema, plaque psoriasis, contact dermatitis, seborrheic dermatitis, localized granuloma annulare, fixed drug eruptions, and subacute cutaneous lupus erythematosus. Hence, proper clinical evaluation and diagnostic testing are crucial in distinguishing these conditions from tinea corporis and ensuring appropriate treatment [12].

Different classes of antifungal drugs are available for treatment, including azoles, allylamines, and benzylamines, which interfere with various steps in the ergosterol biosynthesis pathway. Additionally, some antifungal agents work through mechanisms that do not involve lanosterol 14α-demethylase or SQLE. Like many other microbes, dermatophytes can develop resistance to antifungal treatments after prolonged exposure. This resistance has been observed in *Trichophyton*, the genus most frequently linked to human dermatophytosis, particularly against terbinafine and azoles, which are commonly used to treat these infections. Mutations in the SQLE gene, resulting in amino acid changes in the SQLE protein, have been identified as a cause of terbinafine resistance. Similarly, single nucleotide variations in genes encoding lanosterol 14α-demethylase, along with the overexpression of efflux pump genes, are associated with azole resistance [5].

The treatment of dermatophytosis varies based on its severity and extent, with options ranging from topical to oral antifungal medications, either as monotherapy or in combination. Most cases of tinea corporis can be effectively treated with topical antifungals, which should be prioritized whenever possible due to their safer profile and minimal side effects. However, for widespread infections or cases where topical treatments are ineffective, systemic therapy is required [1].

The preferred treatment for dermatophytosis caused by this pathogen is itraconazole, administered at a dosage of 100 mg twice daily for 4-8 weeks and, in some cases, up to 12 weeks [8]. Although available data are limited, fluconazole and griseofulvin have shown minimal in vitro or clinical effectiveness. Some evidence suggests that certain patients may still benefit from griseofulvin, particularly when administered in higher doses and over extended treatment durations [6].

Combining oral treatment with topical antifungals is always recommended due to their synergistic effect. Azoles such as clotrimazole, miconazole, luliconazole, sertaconazole, bifonazole, and eberconazole, along with ciclopirox and amorolfine, are available in several countries. Luliconazole, a newer topical azole antifungal, has demonstrated superior in vitro efficacy against both zoophilic and anthropophilic dermatophytes, particularly *T. indotineae* [7].

Tinea corporis is a contagious condition that can have considerable psychological, social, and occupational health implications. In some cases, excessive scratching or skin abrasion may lead to secondary bacterial infections. Additionally, post-inflammatory skin changes, such as hypopigmentation or hyperpigmentation, can develop. A dermatophytid reaction, also known as an id reaction, autoeczematization, or disseminated eczema, is a secondary dermatitic eruption that may occur in response to a fungal infection, often shortly after initiating systemic antifungal therapy. This reaction typically presents as widespread, intensely itchy, erythematous, and scaly papules, maculopapules, papulovesicles, or pustules [12].

## Conclusions

This case highlights the potential clinical failure of both systemic and topical terbinafine in treating dermatophytosis, likely due to resistant strains. The observed improvement following the switch to a combination of topical clotrimazole and luliconazole suggests that alternative topical agents may be effective in managing terbinafine-refractory cases. Although susceptibility testing was not available, the patient's favorable response supports the need for further investigation into the role of non-terbinafine antifungals, particularly luliconazole, in treating suspected resistant dermatophyte infections. This case underlines the importance of treatment flexibility and careful monitoring in managing recalcitrant dermatophytosis.
